# Ground golf-induced changes in the blood pressure of healthy elderly people

**DOI:** 10.1186/s40101-020-00220-2

**Published:** 2020-04-05

**Authors:** Naoyuki Ebine, Masahiro Itoh, Masahiro Horiuchi, Tatsuya Hojo, Miho Yoshimura, Yoshiyuki Fukuoka

**Affiliations:** 1 Faculty of Health and Sports Science, Doshisha University, Kyoto, 610-0394 Japan; 2 Kumamoto University Graduate School of Life Sciences, Kumamoto, 862-0976 Japan; 3 Division of Human Environmental Science, Mount Fuji Research Institute, Yamanashi, 403-0005 Japan; 4 Faculty of Environmental and Symbiotic Sciences, Prefectural University of Kumamoto, Kumamoto, 862-8502 Japan

**Keywords:** Ground golf, Elderly, Mean arterial pressure

## Abstract

**Background:**

Ground golf is a popular sport among the elderly in Japan. Several types of exercise can reduce the body's mean arterial pressure (MAP), but little is known about how ground golf affects the MAP. We investigated the effects of ground golf on the MAP and the oxygen uptake ($\dot{\mathrm{V}}{\mathrm{O}}_2$) in a healthy elderly population.

**Participants and methods:**

Thirteen elderly Japanese people (3 males and 10 females, mean age of 66 years) participated. All participants played 8 holes of ground golf 6 times, as game (G)1 to G6. The MAP, heart rate (HR), and $\dot{\mathrm{V}}{\mathrm{O}}_2$ were measured at rest and every 5 min during each game.

**Results:**

A linear trend analysis revealed that participants’ MAP values progressively decreased as each game proceeded with marginal differences (*p* = 0.054). There were no significant differences in HR between at rest and any of the games. The $\dot{\mathrm{V}}{\mathrm{O}}_2$ during the games (except for G6) were significantly higher than that at-rest (*p* < 0.05). The resting MAP values were negatively associated with the ground golf-induced changes in MAP (*r* = 0.786, *p* = 0.001). The participants with greater changes in $\dot{\mathrm{V}}{\mathrm{O}}_2$ during the games showed significantly greater reductions in MAP (*r* = 0.276, *p* = 0.043).

**Conclusions:**

Playing ground golf reduced the participants’ MAP and increased their $\dot{\mathrm{V}}{\mathrm{O}}_2$. Participants with higher resting MAP experienced greater reductions in MAP by playing ground golf, which suggests that ground golf can be a useful recreational sport for the elderly.

## Background

**Fig. 1:**
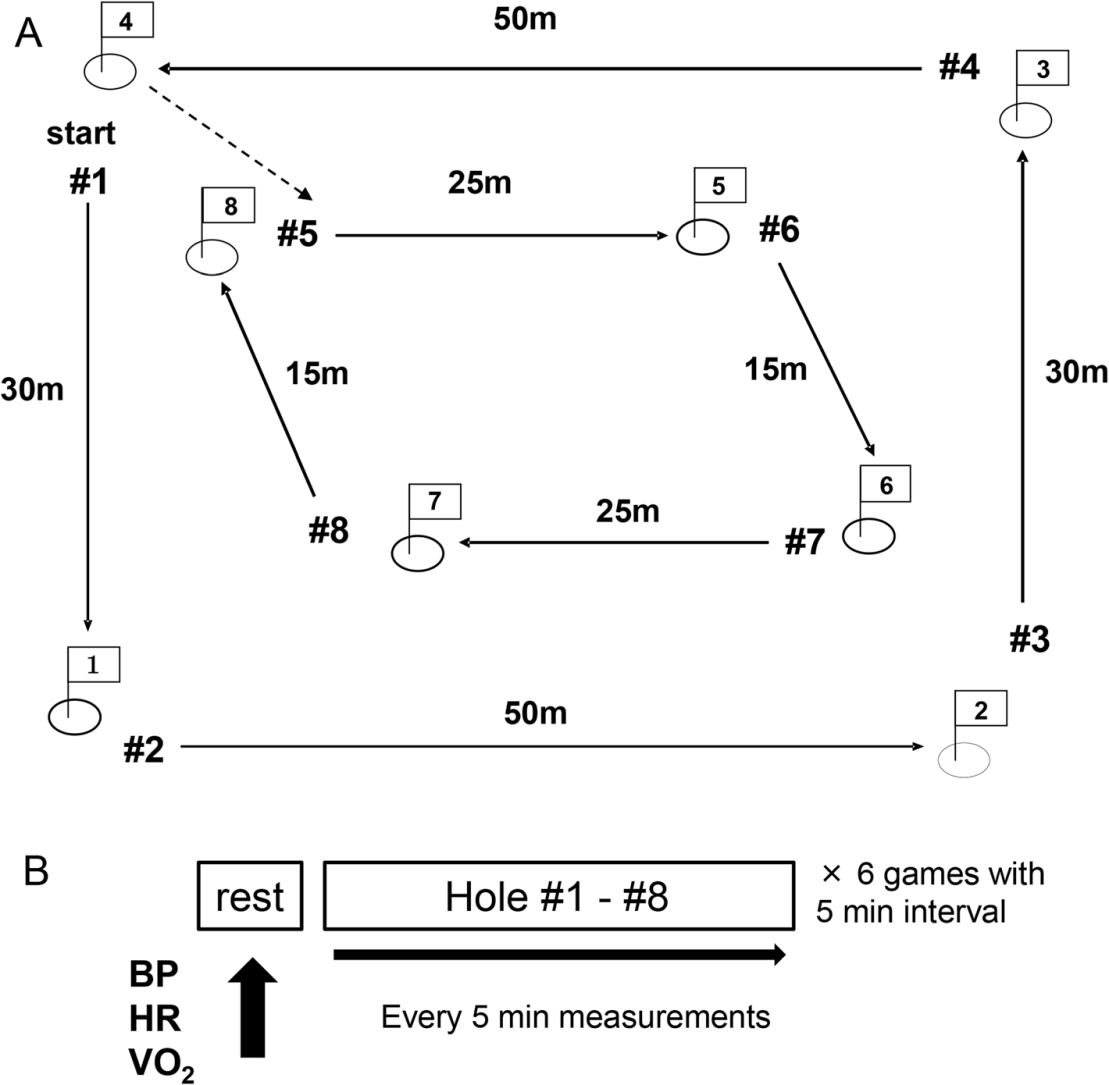
Study procedure. **a** An illustration of ground golf play. One game consists of eight holes. **b** Measurement procedure

**Fig. 2:**
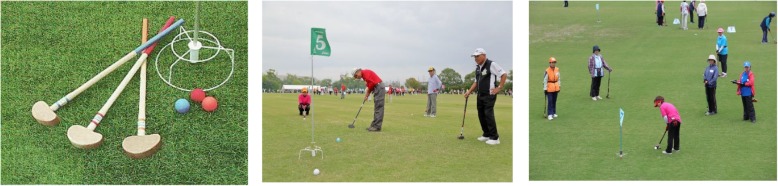
Photographs which the tools of sticks, ball and hole (*left*), and the elderly individuals of ground golf play (*middle*, *right*). From International Ground Golf Federation, with permission

Ground golf is popular among the elderly in Japan; players compete to make the fewest number of strokes over eight holes (Fig. 1a), alternately walking and hitting the ground golf ball (Fig. 2). These non-impact characteristics make ground golf appropriate recreation for elderly individuals [1]. Prolonged or high-intensity exercise may not be appropriate for the elderly, as the risk of hypertension increases with aging [2]. An acute single bout of physical exercise can elicit an ~ 2h reduction of arterial blood pressure (BP) compared to the baseline (pre-exercise) levels [3–5]. Post-exercise hypotension has also been observed after moderate prolonged aerobic exercise [6, 7] and high-intensity resistance exercise [8, 9]. Walking exercise elicited reductions in BP after exercise cessation [10–12].

Little is known about how ground golf affects exercise-induced BP changes. Herein, we tested our hypothesis that in the healthy elderly, playing ground golf would reduce BP compared to the resting baseline BP.

## Participants and methods

### Participants

Three males and 10 females (mean age 66 years, range 59–76) participated. Table 1 summarizes that their physical characteristics and medication status. Three subjects took medicines of hypertension regularly, one of three subjects took medicines of thrombus additionally. All female participants were in menopause. A detailed explanation of the study procedure, possible risks, and benefits of participation was provided. This study was approved by the Prefectural University of Kumamoto’s ethical committee and was in accordance with the Declaration of Helsinki. The participants voluntarily signed informed consents. All participants were non-smokers. They were asked to abstain from caffeinated beverage and alcohol for 12 h and refrain from strenuous exercise for 24 h before the study.

**Table 1:** Individual physical characteristics and medication status

Pt. No.	Sex	Age, years	Height, m	Body mass, kg	BMI, kg (m^2^) ^−1^	Medications
1	M	70	1.67	84	30	
2	M	59	1.68	81	29	Hypertension
3	M	64	1.69	65	23	
4	F	68	–	–	–	
5	F	67	1.50	49	22	Hypertension
6	F	62	1.43	42	21	Hypertension
7	F	74	1.37	33	18	
8	F	64	1.49	57	26	
9	F	72	1.54	65	27	Hypertension, thrombus
10	F	64	1.56	56	23	
11	F	76	1.45	44	21	
12	F	61	1.45	48	23	
13	F	59	1.68	81	29	
Mean		66	1.54	59	24	
SE		1.5	0.03	5	1	

– indicates no data. *BMI* body mass index, *F* female, *M* male, *SE* standard error

### Study protocol

All participants played a game of ground golf (i.e., eight holes) six times as games (G)1 to G6 with 2–3 min intervals between games. The rules and tournament management of ground golf are accessible on the website of the International Ground Golf Federation in detail [13]. The resting baseline BP values at a standing position were measured before G1. During each game, participant’s respiratory gas exchange variables and BP (see below for measurement details) were monitored automatically every 5 min (Fig. 1b).

### Measurements

The participant’s systolic (SBP) and diastolic blood pressure (DBP) were measured every 5 min by a portable automatic monitoring device using oscillometric methods (COLIN ABPM-630, Nippon Colin Co., Tokyo). The heart rate (HR) was measured by the same device. The pulmonary oxygen uptake ($\dot{\mathrm{V}}{\mathrm{O}}_2$) was measured every 1 min with a portable metabolic chart (Mac Quarto; VM4-064, Vine Co., Tokyo). During the measurement, the participant stopped and maintained a quiet standing position. BP and HR were measured once at each point, and $\dot{\mathrm{V}}{\mathrm{O}}_2$ was measured for 1 min at each point. The participant resumed playing after measurements. Due to the limited number of devices, the $\dot{\mathrm{V}}{\mathrm{O}}_2$ was obtained from nine of the 13 participants.

### Data analysis

The mean arterial blood pressure (MAP) was calculated:

$$ \mathrm{MAP}=\left(\mathrm{SBP}-\mathrm{DBP}\right)/3+\mathrm{DBP} $$

The participants’ BP and $\dot{\mathrm{V}}{\mathrm{O}}_2$ were measured every 5 min during each game. It took ~ 12–13 min to complete one game (eight holes). We thus obtained BP and $\dot{\mathrm{V}}{\mathrm{O}}_2$ values 2–3 times during a single game, and the averages of these measurements were used.

### Statistical analyses

All values are presented as the mean ± standard error (SE), which represents a within-participant deviation for estimation of the range for the “true” mean value. We performed one-way repeated-measures ANOVA with a linear trend analysis and pairwise (Dunnett) post hoc tests to evaluate the changes in all physiological variables across the games and resting baseline. The Pearson correlation coefficient was used to evaluate the relationship between the MAP values before the games and during G6, and between the changes in $\dot{\mathrm{V}}{\mathrm{O}}_2$ and the changes in MAP. Probability values < 0.05 were considered significant. The statistical analyses were performed with SPSS (Statistics 25, IBM).

## Results

**Fig. 3:**
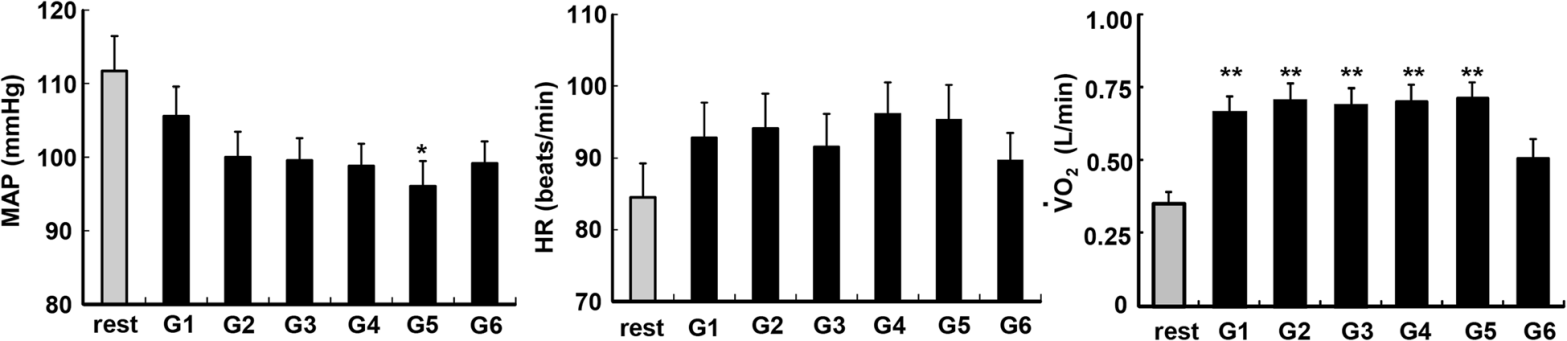
Comparisons of the mean arterial pressure (MAP; *left*), heart rate (HR; *middle*), and pulmonary oxygen uptake ($\dot{\mathrm{V}}{\mathrm{O}}_2$; *right*) during the ground golf game. Values are mean ± standard error (SE). G: game. *p < 0.05, **p < 0.01 vs. at rest

Figure 3 illustrates the changes in MAP, HR, and $\dot{\mathrm{V}}$O_2_ during the six games. The linear trend analysis revealed that participants’ MAP values progressively decreased as the game proceeded, with marginal differences among the values (*p* = 0.054). The MAP (96 ± 3 mmHg) during G5 was significantly lower than that at rest (112 ± 5 mmHg). The overall HR values were slightly higher during the games at ~ 95 bpm versus those at resting baseline (85 ± 5 bpm). The participants’ $\dot{\mathrm{V}}{\mathrm{O}}_2$ during the games (range 0.66–0.71 L min^−1^) was twice higher than that at the resting baseline (0.35 ± 0.03 L min^−1^) with significant differences between the games (G1 to G5) and baseline (all *p* < 0.05) except between G6 and rest (*p* > 0.05).

**Fig. 4:**
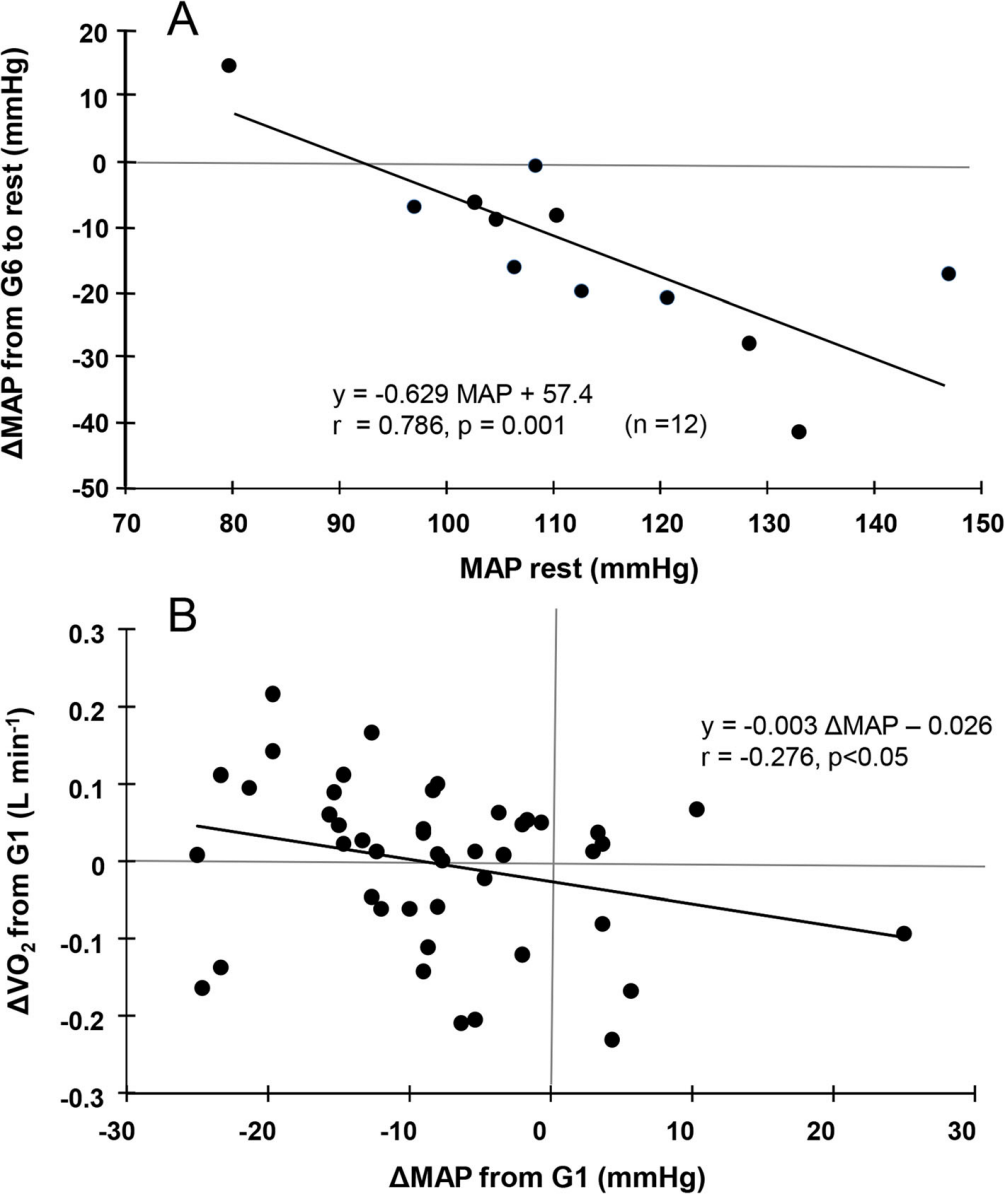
**a** The relationship between resting MAP values and ground golf-induced changes in MAP (the difference between the values of MAP at G6 and rest). Note that the data of one participant (#8) could not be obtained; the sample size was 12. **b** The relationship between ground golf-induced changes in MAP and $\dot{\mathrm{V}}{\mathrm{O}}_2$. Note that $\dot{\mathrm{V}}{\mathrm{O}}_2$ was obtained from nine participants; the total sample size was 45 (9 participants × 5 game points)

Figure 4 illustrates the relationships between the participants’ resting baseline MAP values and averaged MAP values during G6, and the changes in MAP and $\dot{\mathrm{V}}{\mathrm{O}}_2$ from G1 to G6 when the data are pooled (9 participants × 5 game points = 45). Higher resting MAP was significantly associated with a greater MAP reduction (*r* = 0.786, *p* = 0.001, Fig. 4a). Participants with greater changes in $\dot{\mathrm{V}}{\mathrm{O}}_2$ during the games showed greater MAP reductions (*r* = 0.276, *p* = 0.043, Fig. 4b).

## Discussion

The mechanisms underlying the reductions in MAP during ground golf are unknown. Since BP is determined by a functional product of the cardiac output [HR × stroke volume (SV)] and the total vascular resistance (TVR), it may be reasonable to first focus on these two basic components (three parameters). Herein, since we observed no significant increases in HR, it is thus possible that the decreases in BP variables were attributable to decreases in the SV and/or TVR. Exercise-induced hypotension has been thought to be caused by a decrease in TVR that is incompletely offset by an increase in cardiac output [3–5], but in older hypertensive patients, exercise-induced hypotension is mediated largely by decreased cardiac output [14].

Herein, all of the participants maintained a standing position while they played the ground golf games (including during the BP measurements); cardiovascular responses to orthostatic stress in this elderly population should thus also be considered. In another study, despite the similar orthostatic tolerance between young and older participants, the cardiovascular responses to orthostatic stress were markedly different between the young and older subjects; indeed, the SV was lower in the young subjects, whereas the TVR was lower in the older subjects due to orthostatic stress [15]. The different study settings between these studies [14, 15] and ours along with the lack of SV and TVR data in this study make it difficult to explain the mechanisms underlying reductions in the BP observed herein.

Notably, the present participants with higher resting MAP experienced greater MAP reductions. The participants with a normal BP range did not show similar MAP reductions (Fig. 3a). This finding is of particular concern regarding elderly individuals with hypertension, because ground golf may be an effective sport for reducing elderly hypertensives’ BP. Individuals with higher resting BP values may show greater hypotension after a dynamic exercise [16]. Similar benefits were observed among aged people after they spent time walking in a forest [17]. Therefore, the significant MAP decreases in the present study’s elderly participants can be partly accounted for by the participants’ differing baseline BP values.

Additional issues are the ground golf-induced increase in $\dot{\mathrm{V}}{\mathrm{O}}_2$ and the negative relationship between $\dot{\mathrm{V}}{\mathrm{O}}_2$ and MAP. Here, playing ground golf increased the participants’ VO_2_ by ~ twofold and increased the participants’ HR by ~ 10 bpm compared to rest. Moreover, the participants with a greater increase in $\dot{\mathrm{V}}{\mathrm{O}}_2$ showed greater MAP reductions. A recent study compared the energy expenditure (i.e., $\dot{\mathrm{V}}{\mathrm{O}}_2$) and BP responses among sedentary videogame, active videogame, and walking exercise conditions in hypertensive women (mean age 57 years) [18]. Compared with the sedentary videogame condition, the subjects’ $\dot{\mathrm{V}}{\mathrm{O}}_2$ values were 2–3 times higher than that at rest, and the HR reached an average of ~ 105 bpm in the active videogame condition and walking exercise [18]. Those authors observed a similar magnitude of post-exercise hypotension after active videogame and walking exercises: an ~ 10 mmHg BP reductions [18]. As the HR and BP were similar between that study [18] and ours, we speculate that relatively light-intensity exercise (e.g., ground golf) may provide a sufficient stimulus to reduce BP in elderly populations. Our results also highlight the importance of the exercise intensity for obtaining exercise-induced BP reductions. It was suggested that higher intensity and longer duration cause a greater and longer duration of BP reductions after a single exercise bout [19–21] and after walking in a forest [10].

Our sample size (*n* = 13) is lower than optimal, and this increased the chance of false-negative results (type 2 error) when multiple comparisons were made. However, the linear trend analysis that we used addressed the concern of low statistical power by focusing on the overall slope and fit of the response in BP variables across the six games. This approach was particularly advantageous [22] because MAP changes were subtle across the six games and would have required a larger sample size to detect pairwise differences in the values between the baseline at rest and those obtained during the six games. In relation to the sample size, further studies are required whether physical measurements (height, weight, BMI) or sex difference can be influenced to MAP changes across the six games.

## Conclusion

Our results showed that ground golf reduced the participants’ MAP and increased their VO_2_. The participants with higher MAP experienced greater MAP reductions. These results suggest that ground golf is an effective sport for elderly individuals.

## Supplementary information

**Additional file 1.**

## Data Availability

Blood pressures and oxygen uptake raw data used in this study were summarized and provided in the supplementary information.

## Abbreviations

BP: Blood pressure
SBP: Systolic blood pressure
DBP: Diastolic blood pressure
MAP: Mean arterial pressure
HR: Heart rate
SV: Stroke volume
TVR: Total vascular resistance
$$ \dot{\mathrm{V}}{\mathrm{O}}_2 $$: Oxygen uptake
